# Evaluating a Preventive Services Index to Adjust for Healthy Behaviors in Observational Studies of Older Adults

**Published:** 2010-08-15

**Authors:** Barbara Williams, Paula Diehr, James LoGerfo

**Affiliations:** Health Promotion Research Center, School of Public Health, University of Washington; University of Washington, Seattle, Washington; University of Washington, Seattle, Washington

## Abstract

**Introduction:**

Analysis of outcome measures from nonrandomized, observational studies of people participating or not participating in health programs may be suspect because of selection bias. For example, fitness programs may preferentially enroll people who are already committed to healthy lifestyles, including use of preventive services. Some of our earlier studies have attempted to account for this potential bias by including an ad hoc preventive services index created from the patient's number of earlier clinical preventive services, to adjust for health-seeking behaviors. However, this index has not been validated. We formally evaluated the performance of this preventive services index by comparing it with its component parts and with an alternative index derived from principal component analysis by using the weighted sums of the principal components.

**Methods:**

We used data from a cohort of 38,046 older adults. We used the following variables from the administrative database of a health maintenance organization to create this index: fecal occult blood test, flexible sigmoidoscopy, screening mammogram, prostate cancer screening, influenza vaccination, pneumococcal vaccination, and preventive care office visits.

**Results:**

The preventive services index was positively correlated with each of the following components: colon cancer screening (*r* = .752), screening mammogram (*r* = .559), prostate cancer screening (*r* = .592), influenza vaccination (*r* = .844), pneumococcal vaccination (*r* = .487), and preventive care office visits (*r* = .737). An alternative preventive services index, created by using principal component analysis, had similar performance.

**Conclusion:**

A preventive services index created by using administrative data has good face validity and construct validity and can be used to partially adjust for selection bias in observational studies of cost and use outcomes.

## Introduction

Researchers often use observational study designs to examine the relationship between health care interventions and health care costs. However, one of the challenges of observational studies is that selection bias may influence both the study population and the measured outcomes. For instance, reviewers of the bias in nonrandomized intervention studies found that results of nonrandomized studies sometimes differ from results of randomized studies of the same intervention (1). They concluded that "standard methods of case-mix adjustment do not guarantee removal of bias."

Although selection bias can never be completely eliminated in such analyses, certain steps can be taken to minimize its effects. We recently published several articles (2-5) in which we compared the health care costs of people who did or did not participate in a physical activity benefit offered to Medicare enrollees. Those analyses, which used a retrospective observational cohort design, controlled for covariates from the administrative data that might have influenced the use and cost outcomes. One covariate was a preventive services index. A preventive services index incorporates measures of the prior use of preventive services to describe a person's tendency to use such services. In this article, a preventive services index attempts to account for the self-selected nature of health-oriented people toward health club enrollment and participation. Specifically, we were concerned that people who take an active role in managing their health may use more preventive medical services and may be more likely to enroll in an exercise program, as other researchers have found (6). Such a tendency, rather than the physical activity program itself, could result in lower costs.

Few observational studies adjust for a person's "prevention-seeking behavior." Researchers who examined the use of statins in preventive therapy (7) used clinical and laboratory data in their models to account for "healthy user status." The authors of a study of menopausal hormone therapy (8) suggested a "healthy user effect." Other researchers have used the term (9) to describe a confounding bias that may affect observational studies of drugs, diets, screening procedures, and other health-related behaviors. To our knowledge, no researchers have used an adjustment for healthy users, in the form of an index, to account for the propensity of people to engage in preventive behaviors, especially a physical activity benefit.

We designed this study to evaluate the validity of a previously created preventive services index, which we have used to control for selection bias in observational studies. We examined this index, constructed from the sum of clinical services available in an administrative database, and compared it with an alternative index created with a different approach, using principal components analysis. We examined the relationship between the indexes and health behaviors and cost outcomes and make suggestions for using this previously created preventive services index in nonrandomized research studies.

## Methods

### Study sample

Our study population consisted of members of Group Health Cooperative of Puget Sound (GHC), a large health maintenance organization in Washington State that enrolls nearly 60,000 Medicare beneficiaries. People were eligible for our study if they were aged 65 or older, lived in the community, and were enrolled in GHC between October 1, 1997, and December 31, 2004. All were eligible to use either a fitness program benefit that consisted of membership at a fitness club (Silver Sneakers) or a specially designed physical activity program (EnhanceFitness). The 2 fitness programs are described in detail elsewhere (3,10). In either case, enrollment is triggered when a person either enters or enrolls in a fitness club or goes to an EnhanceFitness class. We constructed an intervention cohort consisting of all members who signed up for the benefit between January 1, 1998, and December 31, 2003, and who had been continuously enrolled at GHC for at least 1 year before enrolling in either fitness program. The date of first enrollment is called the index date. For each person in the intervention cohort, the control group consisted of 3 GHC members who never enrolled in the program and whom we matched by age and sex to each fitness program participant. Controls were assigned the index date of the participant to whom they were matched.

A total of 40,956 seniors met these qualifications. We later excluded 1,400 seniors who lived outside of the 9-county Puget Sound region and were unlikely to participate in a Puget Sound-based fitness program. Of the remaining 39,556 people, we excluded 1,510 because they lacked cost or use data, for a final sample size of 38,046. Institutional review boards at GHC and the University of Washington approved the study protocol.

### Database

GHC administrative data were the source of all use, cost, and patient demographic variables. The database included diagnostic and use information from medical staff, nursing, pharmacy, laboratory, radiology, hospital inpatient, and community health services and a cost for each of those services. It also included a variable "RxRisk," which is a measure of chronic disease burden or comorbidity calculated by GHC for each person on the basis of age, sex, and pharmacy use for the 6 months before the index date (11,12). To control for chronic disease, we also used the presence of a participant on a diabetes or heart registry. Diabetes registry patients had a hospital discharge diagnosis of diabetes, nonfasting plasma glucose level of 200 mg/dL or higher, fasting plasma glucose level of 200 mg/dL or higher, a hemoglobin A1c level of 7.0% or higher, or a prescription for insulin. Heart registry patients had a diagnosis of angina, coronary heart disease, or acute myocardial infarction.

### Preventive services variables

We designed the preventive services index to make use of all data in the administrative databases related to use of clinical preventive services. These data were fecal occult blood testing and flexible sigmoidoscopy for colon cancer screening, mammograms for breast cancer detection, blood testing for prostate cancer screening, an influenza or a pneumococcal vaccination, and visits coded specifically as a preventive visit up to 2 years before the index date. Insurance benefits completely covered costs of the preventive services in the index for all patients.

For colon cancer screening, we created a new variable by combining number of fecal occult blood tests or a pneumococcal test series and flexible sigmoidoscopies for up to 2 years before the index date up to a maximum of 2. For influenza vaccination we constructed another variable by identifying receipt of influenza vaccine up to 2 years before the index date, counting only 1 per year up to a maximum of 2. Similarly, for pneumococcal vaccinations, we constructed a variable by identifying vaccination up to 2 years before the index date and counted only 1 per year up to a maximum of 2. Screening for prostate cancer was determined by identifying blood tests for prostate-specific antigen (PSA) for up to 2 years before the index date. We counted PSAs if they were coded as a screening PSA test. Only 1 per year was counted up to a maximum of 2. We assessed screening for breast cancer by counting screening mammograms up to 2 years before the index date up to a maximum of 2. Finally, we assessed annual exams or preventive visits for counseling by counting visits coded as preventive visits for up to 2 years before the index date up to a maximum of 2.

### Preventive services index

To estimate each person's "prevention-seeking" behavior and to control for selection bias, we used a preventive services index that we created previously. This index used variables available from the GHC administrative database and was the sum of the number of times a person received colon cancer screening (fecal occult blood test or flexible sigmoidoscopy), a screening mammogram, prostate cancer screening, an influenza vaccination, and a pneumococcal vaccination during the 2 years before an index date (range, 0 to 8). If the person had none of the 4 services in the past 2 years, then the preventive services index was the number of annual examinations or preventive visits the person had in the past 2 years (maximum of 2). Two years was chosen as a time frame for creating the index because, although some preventive services are recommended every year (for example, receipt of influenza vaccine or annual examination), other services are recommended less often (for example, pneumococcal vaccination or mammogram). In addition, measuring the services during a 2-year period allows the inclusion of health-conscious people who get preventive services more or less on an annual basis.

### Alternative preventive services index

To determine whether a different weighting of the preventive variables could be more effective than the ad hoc index in accounting for selection bias, we constructed an alternative preventive services index that used principal components analysis, which yields a composite variable that captures much of the information of the preventive variables. The principal components are weighted sums of the original observed items (13). We decided to use all the preventive variables available to us in the administrative database because we believed that an index based on a group of variables reflects patient health-seeking behavior more accurately than an index based on a single variable. Because screening mammograms are available only for women and screening examinations for prostate cancer are available only for men, we created 4 principal component scores to account for the lack of the same variables being available for both sexes. The first alternative index (labeled "men or women") included 4 variables common to both men and women (influenza vaccination, pneumococcal vaccination, preventive visits, and screening for colon cancer) plus a variable that represented the number of mammograms or screenings for prostate cancer. We created a second index using only the 4 variables common to both men and women, which we labeled "men and women." Finally, we created a "men only" index and a "women only" index, each having the 5 behaviors available to each of these sexes.

### Statistical analysis

We used *t* tests and cross-tabulation to examine any differences in the demographic characteristics between men and women in the sample. We used correlation coefficients to describe the relationship between the demographic and use variables and the indexes. We performed principal components analysis  by using the FACTOR command in SPSS version 15.0 for Windows (IBM, Chicago, Illinois). We used pairwise deletion, principal component extraction, correlation method, and no rotation to determine the factor loadings of the components of the alternative preventive services index.

### Analytical approach

The first step of our analysis plan was to determine the relationship between the preventive services index used in earlier publications and the various items of this preventive services index. We then created a new alternative index by using principal components analysis that included the same items found in the original preventive services index but had 4 variations based on the sex of the participant. We examined the relationship between these preventive services indexes and baseline demographics, fitness program enrollment, and attendance data. Finally, we compared the relationship between the original preventive services index, the alternative principal components analysis preventive services index, and age group.

## Results

The mean age of our sample was 73 years; 62% were women (Table 1). Sixteen percent had at least 1 outpatient visit with an International Classification of Diseases (ICD)-9 code for arthritis in the year before the index date. Nineteen percent were on the heart registry and 15% were on the diabetes registry. Twenty-seven percent of the participants were enrolled in the EnhanceFitness program; 22% were in Silver Sneakers. Use of preventive services in the 2 years before the index date was as follows: 40% had an influenza vaccination, 14% had a pneumonia vaccination, 32% had either returned stool cards or had a flexible sigmoidoscopy, 17% of the men had a PSA test, 68% of the women had a mammogram, and 47% had a preventive office visit. The *t* test comparison for the preventive services index between men and women was *t* = 35.1, *df* = 30,913, *P* < .001. The difference between men and women was mostly due to the difference in the frequencies of PSA tests versus mammograms. The mean preventive services index (range, 0-8) was 1.78 (SD, 1.72). The mean annual total per person health care costs for the year before baseline was $5,471 (SD, $10,752), the per person inpatient cost for participants with any inpatient use was $11,209 (SD, $14,541), and the per person annual primary care visit cost was $720 (SD, $851). Although the standard deviations for most of these continuous measures were large and could affect the results, the 95% confidence intervals were small. The 95% confidence interval for RxRisk, for example, was $2,629 to $2,673. So although the variance was large, most people had values that fell within a narrow range in these continuous baseline measures.

The correlation coefficients between the 6 variables are included in the preventive services index and RxRisk (Table 2). All correlations were significant (*P* < .001) and in the expected direction, and the preventive services index was most strongly correlated with influenza vaccination (*r* = 0.844), colon cancer screening (*r* = 0.752), and preventive office visits (*r* = 0.737). People with more medications for chronic conditions (higher RxRisk) were less likely to have preventive procedures.

The second index was the first principal component of the preventive variables. The correlations between the items and the principal component (factor loadings) showed that, as expected, all of the individual items were highly correlated with the 4 newly created alternative preventive services indexes (Table 3). The factor loadings were fairly consistent between the 4 methods used to determine the new score. Influenza vaccinations and preventive office visits had the highest factor loadings.

The various indexes were similarly and significantly correlated with other patient characteristics when grouped by sex (Table 4). As expected, RxRisk and health care costs were negatively correlated with the factor scores, whereas enrollment and attendance in either Silver Sneakers or EnhanceFitness  were positively correlated with the factor scores. The original index (based on the sum of the preventive services) compared well with the indexes derived from factor analyses. The alternative "women only" index had slightly higher correlations than the "men only" index in almost all of the cost and enrollment characteristics, except for household income (Table 4). Both the original preventive services index and the newly created principal component analysis scores showed sensitivity to age; the scores decreased with age (Figure).

**Figure 1.**
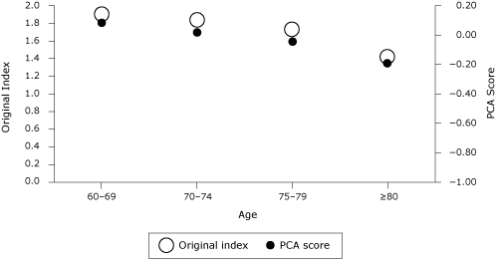
Original preventive services index and principal component analysis score by age group (N = 38,046). Abbreviations: PCA, principal component analysis; PSA test, prostate-specific antigen. Data are from "PCA: Men or Women" in Table 3, where variables are influenza vaccination, pneumoccocal vaccination, colon cancer screening, PSA test, mammogram, and preventive office visit

**Table:** 

**Age, y**	**Original Index**	**PCA Score**
60-69	1.90	0.0820
70-74	1.84	0.0219
75-79	1.74	−0.0254
≥80	1.43	−0.1879

## Discussion

The purpose of this study was to evaluate the preventive services index and its relationship to patient characteristics and to an alternative index based on principal components analysis. Both indexes were highly correlated with preventive behaviors, in the expected direction. The negative correlation with RxRisk may be attributed to clinical health care providers who have less time to address preventive measures in patients with more chronic illnesses (14,15). These indexes were most highly correlated with enrollment in Silver Sneakers or EnhanceFitness.

The alternative preventive services indexes, created by using principal components analysis, performed as well or slightly better than the original index based on the sum of 5 preventive services. The correlation coefficients of the principal components analysis for women only (Table 4) were almost always higher than the correlation coefficients for men, suggesting a closer relationship between the use of preventive services and enrollment in a fitness class for older women.

The observation that both the original and alternative preventive indexes decrease with age implies that the oldest adults were less likely to use preventive services. One explanation for this decrease is that as patients reach the end of life, the focus is no longer on preventive care but on pain management, for example. In addition, some preventive services (colon and prostate screening tests) are no longer recommended for patients beyond a certain age. For example, the PSA test is no longer recommended for men older than 75 years and yet was included in this analysis. This may be one of the limitations of this study.

The original index, a simple arithmetic score, was developed to adjust for possible selection bias reported in studies that found lower subsequent costs for people who took advantage of a physical activity benefit. The cost differential remained significant even after controlling for the preventive services index (B. Williams, unpublished data). In the current analysis, designed to explore the performance of the index, we found that the original index and the variants resulted in similar findings. Because the original preventive services index is easier to calculate than the variants, we recommend its continued use to adjust for this type of selection bias. Thus, this method is generalizable to other research settings in which the sum of clinical services is available from an administrative database.

### Strengths and limitations

One of the strengths of this study is that we were able to use the cost and use database of a large managed care organization, which has a total sample of more than 30,000 people. In addition, the preventive services index does not rely on self-report, which can be subject to error.

Our study had some limitations. We did not know the medical history of the participants, including the presence or absence of a previous cancer diagnosis. We summarized the use of screening (as opposed to diagnostic) colon, mammogram, and prostate examination services, which might not be appropriate for a patient with cancer. For example, our summary score may be high for the estimated 4% of the women older than 60 years who may have had a previous diagnosis of breast cancer or for 1% of the men with a possible previous diagnosis of prostate cancer. Similarly, we did not know how many of the women in our sample had had hysterectomies or mastectomies and might not require a screening test for the corresponding cancers. Furthermore, primary care physicians may influence the use of preventive services by suggesting services to their patients. Patients may choose services based on the recommendations of their physician rather than on their own prevention-seeking initiative. On the other hand, these patients are members of GHC, a health maintenance organization, in which preventive services are encouraged by being offered at no additional cost to the patient.

We were also limited by the variables in the GHC database, and we did not have access to data on other preventive health care services such as cholesterol checks or blood pressure screens. The administrative database does not account for reasons a person might choose not to engage in preventive behavior, including transportation problems, mental status, or physical inability. These variables may have contributed additional information to the preventive services index. Also, because our database was restricted to people who were aged 65 years or older, this analysis may not apply to a younger population.

### Conclusion

Selection bias is a common problem in nonrandomized, observational studies of health care cost and use. We demonstrated that a preventive services index can be easily created from an administrative database to adjust for selection bias in observational studies. An alternative index derived from principal component analysis could be used, but we recommend using the original index because it is simpler to compute. Overall, the index displayed good properties, suggesting its appropriateness to control for selection bias among people who participate in preventive or disease self-management activities. This method may be generalizable to researchers who have access to medical administrative data and need to adjust for selection bias in observational studies.

## Figures and Tables

**Table 1:** Characteristics of Participants at Baseline, Silver Sneakers and EnhanceFitness Programs,^a^ 1997-2004 (N = 38,046)

Characteristics	Total Sample		Men (n = 14,443)		Women (n = 23,603)	
Mean age (SD), y	73.2 (6.0)		73.0 (6.0)		73.5 (5.9)	
Age ≥80, %	16.0		16.9		15.5	
**Women (%)**	62.0		NA		NA	
**Comorbidities**						
RxRisk^b^, $, mean (SD)	2,651 (496 to 4,806)		2,649 (351 to 4,957)		2,652 (590 to 4,714)	
Arthritis, %	16.3		12.9		18.3	
On heart registry, %	19.4		26.2		15.2	
On diabetes registry, %	15.0		18.0		13.2	
**Enrolled in health program^b^**						
Silver Sneakers, %	22.1		24.0		21.0	
EnhanceFitness, %	26.5		26.5		26.4	
**Preventive services index and annual cost measures**						
**Preventive services index,^c^ mean (SD)**	1.78 (1.72)		1.39 (1.67)		2.02 (1.70)	
**Annual cost measures**						
Total health costs, $, mean (SD)	5,471 (10,752)		5,961 (11,961)		5,171 (9,928)	
**Preventive services **	**Mean (SD)**	**%**	**Mean (SD)**	**%**	**Mean (SD)**	**%**
Influenza vaccination	0.67 (0.87)	40.3	0.66 (0.87)	39.5	0.68 (0.88)	40.8
Pneumococcal vaccination	0.14 (0.35)	14.0	0.14 (0.35)	13.7	0.14 (0.35)	14.2
Colon cancer screening	0.38 (0.61)	31.5	0.38 (0.61)	31.4	0.38 (0.60)	31.5
PSA test	0.19 (0.45)	16.5	0.19 (0.45)	16.5	NA	NA
Mammogram	0.79 (0.62)	68.2	NA	NA	0.79 (0.62)	68.2
Preventive office visits	0.56 (0.66)	47.2	0.54 (0.67)	45.5	0.58 (0.66)	48.3

Abbreviations: SD, standard deviation; NA, not applicable; PSA, prostate-specific antigen.

a Silver Sneakers and EnhanceFitness are fitness programs. Silver Sneakers is a paid membership at a fitness club; EnhanceFitness is a specially designed physical activity program.

b RxRisk is a measure of comorbidity and is calculated by Group Health Cooperative for each person based on age, sex, and pharmacy use for the 6 months before the index date.

c Preventive services index is the total number of preventive services that a person used in the 2 years preceding the index date (colon cancer screening [fecal occult blood test or flexible sigmoidoscopy], screening mammogram, prostate cancer screening, influenza vaccination, or pneumococcal vaccination) (range, 0-8).

**Table 2:** Correlation Coefficients Between Original Preventive Services Index and RxRisk,^a^ and Component Index Variables, Silver Sneakers and EnhanceFitness Programs, 1997-2004 (N = 38,046)^b^

Preventive Services in Past 2 Years	Influenza Vaccination	Pneumococcal Vaccination	Colon Cancer Screening	PSA	Mammo- gram	Preventive Office Visit	Preventive Services Index
Pneumococcal vaccination	0.310						
Colon cancer screening	0.549	0.255					
PSA (n = 14,443)	0.337	0.200	0.316				
Mammogram (n = 23,603)	0.237	0.086	0.199				
Preventive office visit	0.688	0.359	0.554	0.518	0.214		
Preventive services index^c^	0.844	0.487	0.752	0.592	0.559	0.737	
RxRisk	−0.047	−0.066	−0.074	−0.090	−0.038	−0.113	−0.083

Abbreviations: PSA, prostate-specific antigen.

a RxRisk is expressed as predicted 6-month costs and is a measure of comorbidity based on age, sex, and pharmacy use for the 6 months before enrollment in either fitness program.

b The Pearson correlation was used to calculate *P* values*; P* was significant at <.001 for all correlations.

c Preventive services index is the total number of preventive services that a person used in the 2 years before the index date (colon cancer screening [fecal occult blood test or flexible sigmoidoscopy], screening mammogram, prostate cancer screening, influenza vaccination, or pneumococcal vaccination) (range, 0–8).

**Table 3:** Factor Loadings for the Principal Component Analysis for Components of Preventive Services Index,^a^ Silver Sneakers and EnhanceFitness Programs, 1997-2004

**Preventive Services in Past 2 Years**	PCA: Men or Women (N = 38,046)	PCA: Men and Women (N = 38,046)	PCA: Men Only (n = 14,443)	PCA: Women Only (n = 23,603)
Influenza vaccination	0.844	0.855	0.824	0.850
Pneumococcal vaccination	0.535	0.554	0.532	0.531
Colon cancer screening	0.767	0.779	0.758	0.768
PSA test (n = 14,443)	0.434	NA	0.630	NA
Mammogram (n = 23,603)		NA	NA	0.393
Preventive office visits	0.866	0.870	0.885	0.859

Abbreviations: PCA, principal component analysis; PSA, prostate-specific antigen test; NA, not applicable.

a Variables for PCA: men or women — influenza vaccination, pneumococcal vaccination, colon cancer screening, PSA test, mammogram, preventive office visit; for PCA: men and women — influenza vaccination, pneumococcal vaccination, colon cancer screening, preventive office visit; for PCA: men only — influenza vaccination, pneumococcal vaccination, colon cancer screening, PSA test, preventive office visit; for PCA: women only — influenza vaccination, pneumococcal vaccination, colon cancer screening, mammogram, preventive office visit.

**Table 4:** Correlation Coefficients of the Original Preventive Services Index or Principal Component Analysis Score, With Other Participant Characteristics, by Sex, Silver Sneakers and EnhanceFitness Programs, 1997-2004

**Component**	Original Index (n = 38,046)	Original: Men Only (n = 14,443)	**Original: Women Only (n = 23,603)**	PCA: Men Only (n = 14,443)	PCA: Women Only (n = 23,603)
RxRisk^a^	−0.083	−0.086	−0.084	−0.099	−0.103
ED baseline costs	−0.082	−0.064	−0.093	−0.068	−0.091
Total baseline costs	−0.068	−0.059	−0.066	−0.065	−0.072
Household income^b^	0.074	0.081	0.079	0.082	0.075
Enrolled in SS or EF	0.187	0.160	0.209	0.154	0.187
SS or EF visits^c^	0.027	0.015	0.064	0.017	0.055
SS or EF months	0.066	0.066	0.073	0.068	0.071

Abbreviations: PCA, principal component analysis; ED, emergency department; SS, Silver Sneakers; EF, EnhanceFitness.

a RxRisk is expressed as predicted 6-month costs and is a measure of comorbidity based on age, sex, and pharmacy use, for the 6 months before enrollment in either fitness program.

b Census tract median household income.

c Total number of SS or EF visits attended (or months of follow-up) during the first year after enrollment for seniors who were either SS or EF enrollees (n = 10,090).
